# Embryogenic cell suspensions for high-capacity genetic transformation and regeneration of switchgrass (*Panicum virgatum* L.)

**DOI:** 10.1186/s13068-019-1632-3

**Published:** 2019-12-16

**Authors:** Christine A. Ondzighi-Assoume, Jonathan D. Willis, Wilson K. Ouma, Sara M. Allen, Zachary King, Wayne A. Parrott, Wusheng Liu, Jason N. Burris, Scott C. Lenaghan, C. Neal Stewart

**Affiliations:** 1 Department of Agricultural and Environmental Sciences, Tennessee State University, Nashville, TN 37209 USA; 2 Department of Plant Sciences, University of Tennessee, Knoxville, TN 37996 USA; 3 Institute of Plant Breeding, Genetics& Genomics, University of Georgia, Athens, GA 30602-7272 USA; 4 Department of Food Science, University of Tennessee, Knoxville, TN 37996 USA; 5 Department of Horticultural Science, North Carolina State University, Raleigh, NC 27607 USA; 6 Center for Agricultural Synthetic Biology, University of Tennessee Institute of Agriculture, Knoxville, TN 37996 USA

**Keywords:** *Agrobacterium tumefaciens*, Bioenergy, Cell suspension culture, Genetic engineering, Switchgrass, Transformation

## Abstract

**Background:**

Switchgrass (*Panicum virgatum* L.), a North American prairie grassland species, is a potential lignocellulosic biofuel feedstock owing to its wide adaptability and biomass production. Production and genetic manipulation of switchgrass should be useful to improve its biomass composition and production for bioenergy applications. The goal of this project was to develop a high-throughput stable switchgrass transformation method using *Agrobacterium tumefaciens* with subsequent plant regeneration.

**Results:**

Regenerable embryogenic cell suspension cultures were established from friable type II callus-derived inflorescences using two genotypes selected from the synthetic switchgrass variety ‘Performer’ tissue culture lines 32 and 605. The cell suspension cultures were composed of a heterogeneous fine mixture culture of single cells and aggregates. *Agrobacterium tumefaciens* strain GV3101 was optimum to transfer into cells the pANIC-10A vector with a hygromycin-selectable marker gene and a *pporRFP* orange fluorescent protein marker gene at an 85% transformation efficiency. Liquid cultures gave rise to embryogenic callus and then shoots, of which up to 94% formed roots. The resulting transgenic plants were phenotypically indistinguishable from the non-transgenic parent lines.

**Conclusion:**

The new cell suspension-based protocol enables high-throughput *Agrobacterium*-mediated transformation and regeneration of switchgrass in which plants are recovered within 6–7 months from culture establishment.

## Background

Switchgrass (*Panicum virgatum* L.) is a perennial C4 prairie grass indigenous to North America, with particular promise as second-generation cellulosic biofuels crop [1–6]. Switchgrass is widely adapted to eastern North America, has low nutrient requirements, high water conversion efficiency, low production costs, and is harvested and stored using established forage practices [2, 7–9]. Although switchgrass has high potential as a bioenergy feedstock, genetic transformation is still inefficient, and breeding is complicated by its perennial habit and polyploidy. Transformation may be required to endow several key sustainability traits, especially cell wall traits needed for reducing recalcitrance for biofuel production [10]. For this reason, efficient and reproducible stable genetic transformation systems are required for genetic improvement of switchgrass. Recently, in vitro methods for genetic engineering have been reported providing opportunities for assaying genes of interest, whereby useful traits have been introduced in few switchgrass genotypes [10–23].

Switchgrass tissue culture and transformation have mainly been constrained to the lowland tetraploid varieties ‘Alamo’ and ‘Performer’ [11, 12, 18, 19, 22–24]. Despite our ability to routinely transform switchgrass, it is a time-consuming and laborious task, which is hampered by low transformation efficiency and copious tissue culture requirements. Switchgrass is considered to be recalcitrant for genetic transformation and depends on the ability of explants (cells or tissues) to regenerate whole plants in culture. Therefore, switchgrass is a good candidate species for biotechnological innovations vis-à-vis cell biology and genetics.

Switchgrass cell suspension cultures, mainly obtained from ‘Alamo,’ have proven to be useful for both cellular research and transformation purposes [11, 25–28]. In these cases, in vitro-cultured immature inflorescences have mainly been used as the source to produce embryogenic callus. The embryogenic callus was then converted to cell suspension cultures. Once in culture, cells and cell clusters will develop into various morphotypes that Mazarei et al. [27] characterized using electron microscopy. Perhaps the most interesting of these, from a biotechnological perspective were the “fine milky type” cultures that consisted of low frequency of single cells and a higher frequency of small cell clusters, which were also amenable to protoplast isolation. However, improvement is needed in the initiation, establishment, maintenance, and applications of switchgrass cell suspension cultures.

*Agrobacterium*-mediated transformation has been successfully used to transform many monocot crops [29, 30], including maize (*Zea mays* L.), wheat (*Triticum aestivum* L.), sorghum (*Sorghum bicolor* L.), barley (*Hordeum vulgare* L.), rice (*Oryza sativa* L.) and Chinese silver grass (*Miscanthus sinensis* A.) and dicots such as cotton (*Gossypium hirsutum* L.), and soybean (*Glycine max* L. Merr.). Indeed, transformation of monocots has progressed in recent years [12, 18, 20, 24, 31–39], which includes tissue culture-based *Agrobacterium tumefaciens*-mediated transformation of switchgrass (*Panicum virgatum* L.) [12, 18, 24]. Li and Qu [18] reported up to 90% transformation efficiency using a ‘Performer’ callus culture. In spite of this achievement, the methodology takes 12 months, and it is laborious.

In this paper we describe significant improvements in throughput and efficiency of transgenic switchgrass production. Our objectives were to: (1) develop a novel embryogenic cell suspension cultures amenable for genetic transformation and regeneration, (2) employ *Agrobacterium*-mediated transformation and accelerated efficient regeneration of transgenic plants.

## Results

### Cell suspension culture characterization

**Fig. 1:**
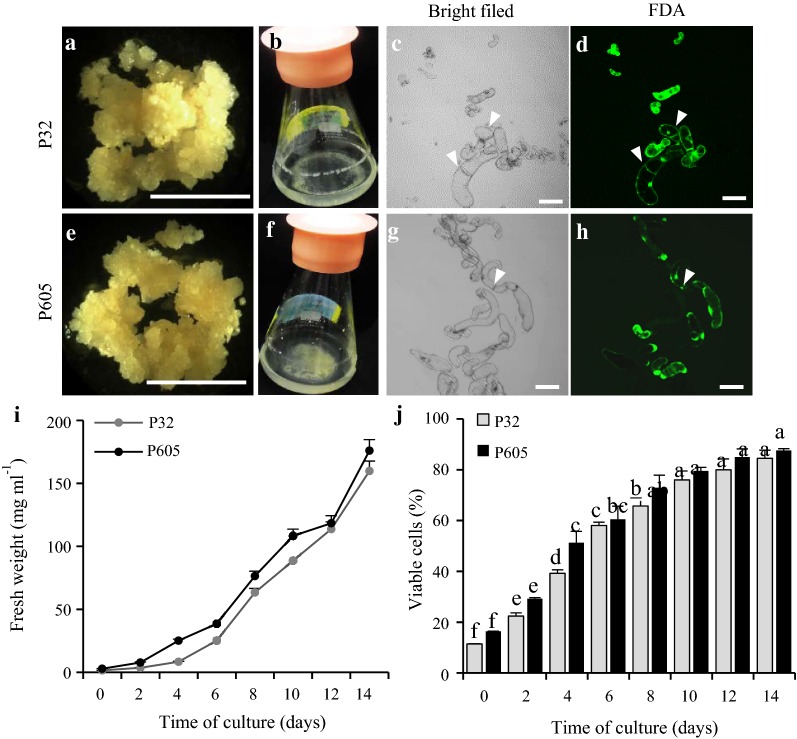
Growth characteristics of P32 and P605 cell suspension cultures. **a** and **e** Images of 1-month-old type II friable embryogenic calli-derived inflorescences of P32 (**a**) and P605 (**e**). **b**, **f** Flasks containing 7-day-old P32 and P605 cell suspension cultured in MSO medium, respectively. **c**–**h** Laser scanning confocal micrographs of viable single and clustered cells stained with FDA in green. **c**, **g** Bright-field micrographs of P32 and P605 cells, respectively. **d**, **h** FDA staining micrographs of P32 and P605 cells, respectively. **i**, **j** Cell density and viability as evaluated with fresh weight (FW) and FDA staining, respectively. Gray circle graph represents P32 cell density. Black circles graph represents P605 cell density. Gray columns represent the percentage of viable P32 cells. Black columns represent the percentage of viable P605 cells. Experiments were done in triplicate. Error bars represent the mean ± standard error (SE). Different letters denote a statistically significant difference among means at a *p* value < 0.05 according to one-way ANOVA (Tukey’s test). White arrowhead indicated dividing cells. Bars = 0.5 cm in **a**, **e**; 50 µm in **c**–**h**

The type of explant and growth parameters are important to achieve reproducible cycles of cell suspension cultures for either laboratory or industry experiments. Thus, growth characteristics for both ‘Performer’ P32 and P605 cell suspension culture lines were analyzed by measuring two different parameters: cell viability and cell density over time. Viable cells were determined by microscopic analysis of cells after fluorescein diacetate (FDA) staining. Viability was measured every 2 days over the course of a 14 days culture period. Up to 79.50 ± 1.73% viable cells grew well in MSO medium by 10 days of culture (Fig. 1c, d, g, h, j), reaching up to 87.60 ± 1.15% by day 14. Using the fresh weight (FW) of cells as a parameter, we found that P32 and P605 cell suspension cultures displayed an increase of the density of cells over 14 days of the culture, reaching the growth phase by 6 days of culture (Fig. 1i). Up to 159.82 ± 1.77 mg ml^−1^ and 174.01 ± 2.32 mg ml^−1^ fresh weight cells were obtained from both P32 and P605 cultures, respectively, after 14 days. Additionally, using the loss of weight by dissimilation (LWD) [40] of P32 and P605 cell suspension cultures over 14 days, we found that this dissimilation was elevated over the time (Additional file 1: Figure S1), indicating that these two cell lines underwent cell division and/or enlargement. The loss of fresh weight by dissimilation is a non-invasive method that allowed us to characterize the growth of both cell suspension cultures by using a single flask without harvesting cells. Control flasks containing only the medium were used to correct losses from water evaporation. Cell morphology of established P32 and P605 cell suspension cultures consisted of heterogeneous mixtures of single oval or elongated cells as well as cell aggregates undergoing active cell division (Fig. 1c, d, g, h).

### *Agrobacterium tumefaciens*-mediated transformation

P32 and P605 embryogenic cell suspension cultures were transformed with *A. tumefaciens* harboring the pANIC-10A expression vector that contains a switchgrass ubiquitin promoter-reporter gene *PvUbi1:pporRFP* and the hygromycin B phosphotransferase II gene (*HYG*). We optimized procedures by using different strains and titer of *A. tumefaciens* (GV3101, GV2260, EHA105, and GV3850) to transform approximatively 88 mg ml^−1^ cell suspension cultures per replicate. The results revealed that only 1 ml of P32 cells aliquoted onto an MSMO plate led to the selection of 1225 ± 1.78 hygromycin-resistant callus sectors expressing the OFP reporter gene (Table 1). Similarly, 1 ml of P605 cells led to an average of up to 1520 ± 0.28 transgenic callus clusters (Table 1). The transformation efficiency varied significantly based on the *Agrobacterium* strain. The highest transformation efficiencies were obtained using GV3101 at 0.5 OD_600_. GV2260 and EHA105 performed moderately well with 820 ± 1.66 and 435 ± 2.89, and 1040 ± 0.67 and 623 ± 0.76 for P605, respectively. GV3850-mediated transformation was ineffective, leading to no transgenic callus for P32 and had an efficiency of only 2.5 ± 2.29% for P605 (Table 1, Additional file 2: Figure S2b). The GV3101, GV2260 and EHA105 strains were more effective than GV3850 in producing more hygromycin-resistant calli, with average efficiencies of 68.47 ± 3.78% vs. 84.42 ± 2.48%, 54.66 ± 5.66% vs. 57.63 ± 4.47%, and 30.76 ± 2.89% vs. 42.85 ± 2.5% compared to 0.0 ± 00% vs. 2.5 ± 2.29% in both P32 and P605 clones, respectively (Table 1). However, GV3101, GV2260 and EAH105 were also effective at the 1.0 OD_600_, but less than at 0.5 OD_600_ in producing hygromycin-resistant calli with average efficiencies varying between 56.7 ± 1.46% vs. 46.67 ± 1.75%, 41.79 ± 0.78% vs. 50.23 ± 0.76% and 20.33 ± 0.70% vs. 31.33 ± 0.76% for both the P32 and P605 clones, respectively (Additional file 2: Figure S2a).

**Table 1:** Transformation efficiencies of *Agrobacterium tumefaciens*-mediated transformation of P32 and P605 cell suspension cultures

Bacterial strain	Selection gene	Reporter gene	P32			P605		
			Total no. of calli grown	Total no. of fluorescent calli	Percentage efficiency (%)	Total no. of calli grown	Total no. of fluorescent calli	Percentage efficiency (%)
GV3101	*HYG*^*R*^	*pporRFP*+	1900 ± 0.07	1225 ± 1.78	68.47 ± 3.78^b^	2128 ± 1.38	1520 ± 0.28	84.42 ± 2.48^a^
GV2260	*HYG*^*R*^	*pporRFP*+	1503 ± 0.03	820 ± 1.66	54.66 ± 5.66^b^	1977 ± 0.33	1040 ± 0.67	57.63 ± 4.47^b^
EHA105	*HYG*^*R*^	*pporRFP*+	1338 ± 0.6	435 ± 2.89	30.76 ± 2.89^c^	1454 ± 0.86	623 ± 0.76	42.85 ± 2.58^c^
GV3850	*HYG*^*R*^	*pporRFP*+	05.00 ± 00	00.00 ± 00	0.00 ± 00^d^	390 ± 0.17	156 ± 1.76	2.5 ± 2.29^d^

Transformation efficiency was calculated for lines of P32 and P605 cell suspension cultures using *Agrobacterium* strains GV3101, GV2260, EHA105 and GV3850 (OD_600_ = 0.5). Transformation efficiency was evaluated by scoring growing hygromycin B (*HYG* ^*R*^)-resistant calli and expressing the fluorescent pporRFP protein. Data are mean ± SD of three replications of transformation events (*n *= 10 plates scored per transformation event for each *A. tumefaciens* strain and per each line). Different letters denote statistically significant differences among means at a *p* value < 0.05 according to one-way ANOVA (Tukey’s test)

### Characterization of transformed cultures

**Fig. 2:**
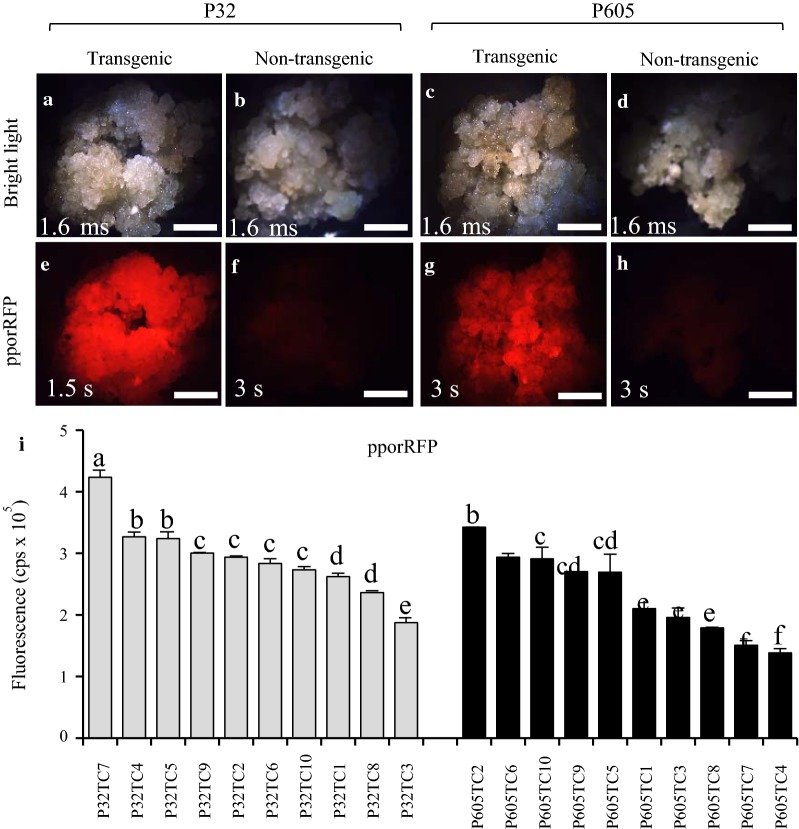
Characterization of stable transgenic P32 and P605 calli expressing pporRFP fluorescent fusion protein. **a**–**h** Micrographs of 1-month-old pporRFP transgenic and non-transgenic (*Agrobacterium* GV3101 harboring no construct) in clones P32 and P605 callus. **a**–**d** Bright-field images of P32 (**a**, **b**) and P605 calli (**c**, **d**). **e**–**h** PporRFP fluorescence images of P32 (**e**, **f**) and P605 (**g**, **h**) calli. **a**, **e** Transgenic P32 calli. **b**, **f**, Non-transgenic P32 calli. **c** and **g** Transgenic P605 calli. **d**, **h** Non-transgenic P605 calli. **i** Graph of pporRFP fluorescence intensity measurements plotted as count per second (cps × 10^5^). Ten independent stable transgenic P32 (gray columns) and P605 (black columns) calli were used. Each column represents the average fluorescence intensity measured from three independent callus pieces (*n* = 3 for each line) at the pporRFP peak emission wavelength (591 nm). All fluorescent measurements were normalized to the non-transgenic calli control. Error bars represent the mean ± SE of three biological replicates, and different letters denote a statistically significant difference among means at a *p* value < 0.05 according to one-way ANOVA (Tukey’s test). Bars = 2 mm in **a–h**

The 30 transformed callus pieces tested for the presence of pporRFP exhibited a bright orange fluorescence; among those calli tested, we found that more transgenic P32 callus had brighter orange fluorescence than the P605 lines (Fig. 2e compared to 2g and 2i). Orange fluorescence was undetectable in non-transgenic control callus (Fig. 2b, f, d, h). Transformed cell suspension culture had very bright orange pporRFP fluorescence as seen under the tdTomato filter set (535–590 nm excitation and 600–650 nm bandpass emission). No pporRFP autofluorescence was observed in cells under DAPI or FITC filter set (Additional file 3: Figure S3). The number of stable transformed calli-derived from liquid cell cultures was found to be correlated to the viability of cells for each clone (P32 or P605) cultured over the time (Additional file 4: Figure S4). The percentage of transformed orange fluorescent- and FDA-viable cells increased over time reaching 90.04 ± 0.68% and 86.5 ± 3.18% (for P32) and 93.93 ± 4.40% to 90.6 ± 0.70% (For P605) by d 14, respectively (Additional file 4: Figure S4a). It is interesting to note that pporRFP co-localized with FDA as we expected both to be cytosolic. This further confirmed the transfer of the foreign gene into the cells (Additional file 4: Figure S4b–i). Stably transformed switchgrass cell suspension cultures of both clones P32 and P605 were maintained and used for the production of transgenic plantlets.

### Organogenesis and regeneration

**Fig. 3:**
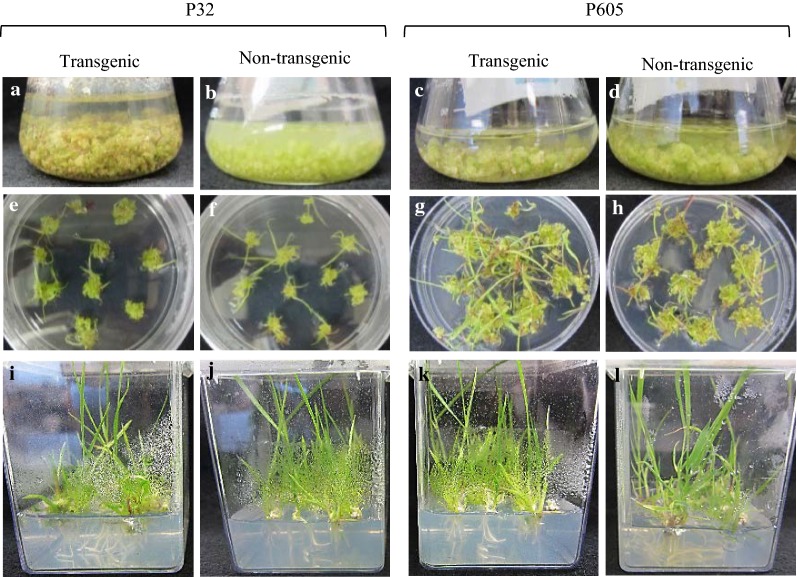
Growth and regeneration of shoots and plantlets from stable transgenic and non-transgenic P32 and P605 callus and cell suspension cultures. **a**–**d** Photos of 2- to 3-week-old regenerated transgenic and non-transgenic P32 (**a**, **b**) and P605 (**c**, **d**) green micro-calli in liquid cultures grown from stable transgenic and non-transgenic cell suspension cultures in REG medium. **e**–**h** Photos of 2-week-old regenerated transgenic and non-transgenic P32 (**e**, **f**) and P605 (**g**, **h**) green multiple shoots grew from green micro-calli cultures in REG solid medium. **i**, **l** Photos of 4- to 6-week-old regenerated transgenic and non-transgenic P32 (**i**, **j**) and P605 (**k**, **l**) plantlets cultured in MSO solid medium

After transfer to regeneration medium, cell cultures initiate shoots as early as 2–3 weeks (Fig. 3a–d). While there is apparent genotype dependency, up to 100 ± 00% of callus produced shoots (Fig. 3e–h, Table 2). In the best cases, up to 91.5 ± 2.11% of the transgenic shoots produced roots compared with 93.5 ± 3.75% rooting of the non-transgenic shoots for P32 (Table 2). Up to 95.28 ± 1.86% to 100 ± 00% rooted plantlets that were transferred to soil developed into plants (Fig. 3i–l, Table 2). For the solidified medium-grown callus, approximately 62.25 ± 1.15 (for P605) to 79 ± 3.51% (P32) of micro-calli differentiated into green shoots, and up to 58.75 ± 0.76 to 74.05 ± 2.35% developed into rooted plantlets (Additional file 5: Figure S5). Also, P605 transgenic callus seldom led to green micro-calli resulting in low frequency of subsequent shooting: 62.25 ± 1.15%, (Table 2).

**Table 2:** Frequencies of shooting, rooting, and viable plant regeneration (mean ± SD) from three replicated experiments starting from 100 micro-calli per line

	Shooting efficiency (%)	Rooting efficiency (%)	Plants regenerated (%)
Regeneration efficiencies of plantlets from calli			
Transgenic P32	73.75 ± 2.92^cd^	69.15 ± 1.35^de^	81 ± 4.88^bc^
Non-transgenic P32	79 ± 3.51^bc^	74.05 ± 2.35^cd^	85.5 ± 1.42^b^
Transgenic P605	62.25 ± 1.15^e^	58.75 ± 0.76^e^	60.25 ± 1.73^de^
Non-transgenic P605	71.67 ± 1.73^cd^	66.25 ± 2.41^de^	75.13 ± 2.00^c^
Regeneration efficiencies of plantlets from cell suspension cultures			
Transgenic P32	100 ± 00^a^	91.5 ± 2.11^a^	100 ± 00^a^
Non-transgenic P32	100 ± 00^a^	93.5 ± 3.75^a^	99.23 ± 0.43^a^
Transgenic P605	100 ± 00^a^	85 ± 2.89^b^	95.28 ± 1.86^a^
Non-transgenic P605	100 ± 00^a^	88.5 ± 0.82^b^	97.78 ± 1.30^a^

After shooting, the rooting percentage represents the frequency of shoots that produced root systems. After rooting, the percentage of regenerated plants represents the frequency of the rooted shoots that survived to make viable plants in pots. Different letters denote a statistically significant difference among means at a *p* value < 0.05 according to a 2-way ANOVA (Tukey’s test)

### Molecular analysis of *T*_0_ P32 and P605 plants

**Fig. 4:**
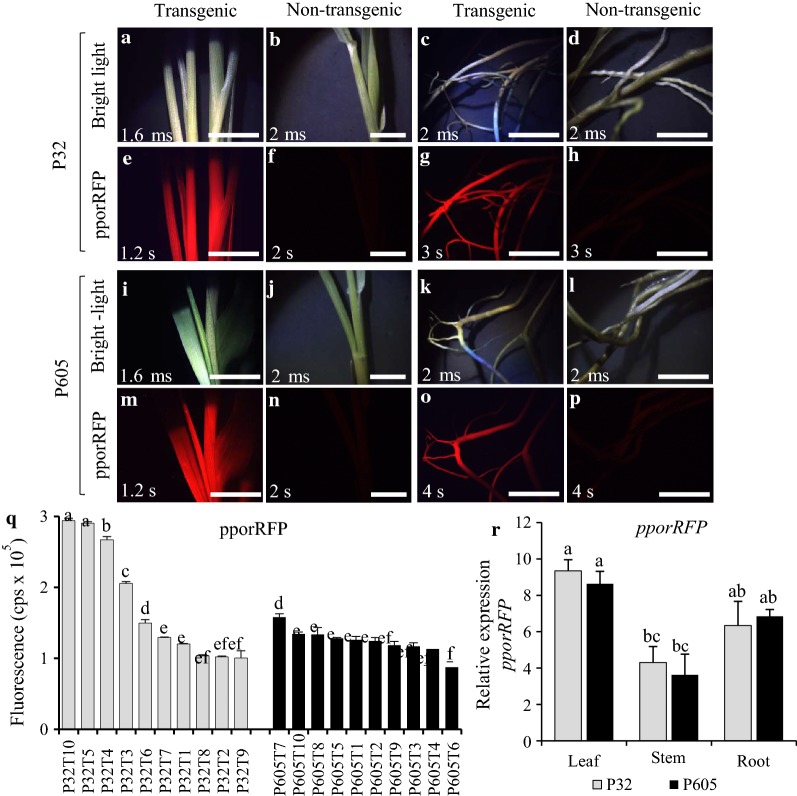
Characterization of regenerated T_0_ P32 and P605 plants expressing *pporRFP*. **a**–**p** Stereomicroscope images showing the presence of pporRFP fluorescence signal in 3-month-old transgenic and non-transgenic P32 and P605 plants regenerated from stable transgenic and non-transgenic single-cell suspension cultures. **a**–**d** Bright-field images of P32 leaves/stems (**a**, **b**) and roots (**c**, **d**). **e**–**h** PporRFP fluorescent images of P32 leaves/stems (**e**, **f**) and roots (**g**, **h**). **i**–**l** White light images of P605 leaves/stems (**i**, **j**) and roots (**k**, **l**). **m**–**p** PporRFP fluorescent images of P605 leaves/stems (**m**, **n**) and roots (**o**, **p**). **q** Graph of pporRFP fluorescence intensity measurements plotted as count per second (cps × 10^5^) of 10 independent transgenic P32 (gray columns) and P605 (gray columns) plants. Fluorescence intensity was measured from youngest fully developed leaves of 10 individual T_0_ plants of each line. Each column (*n* = 3 leaves) represents the average fluorescence intensity at the peak emission wavelength of pporRFP (591 nm). All fluorescent measurements were normalized to the non-transgenic control plants. **r** Expression of reporter gene *pporRFP* in leaves, stems and roots of *T*_0_ transgenic P32 (gray columns) and P605 (black columns) as revealed by qRT-PCR. Error bars represent the mean ± SE of three biological replicates, and different letters denote a statistically significant difference among means at a *p* value < 0.05 according to one-way ANOVA (Tukey’s test). Bars = 0.5 cm. in **a**–**p**

To determine the transgenic status of the first generation of plants (*T*_0_) regenerated from single-cell suspension cultures, the integration, stability, and expression of inserted transgenes into the genome of putative *T*_0_ P32 and P605 plants were analyzed (Additional file 6: Figure S6a–d). PCR analysis of six individual putative *T*_0_ and non-transgenic control plants shown that all *T*_0_ plants generated from the transformation event contained both *HYG B* and *pporRFP* transgenes, indicating that they were transgenic plants. Amplification of the two transgene fragments was not detected in non-transgenic control plants (Additional file 6: Figure S6e). Supporting results obtained with the stereomicroscope and fluorescence spectrophotometry (Fluorolog) systems shown that among ten individual transgenic plants tested, all displayed a bright orange fluorescence in leaves, stems and roots compared with the non-transgenic control plants (Fig. 4a–p), which was congruent with our PCR results. The fluorescence intensity measured in youngest fully developing leaves tissue of the same plants was tenfold higher in both transgenic lines compared with non-transgenic lines. However, the highest intensity was observed in P32 leaves compared to P605 indicating that the pporRFP protein is more highly expressed in P32 leaves (Fig. 4q). Additionally, qRT-PCR showed that both *T*_0_ P32 and P605 plants displayed similar levels of *pporRFP* expression in leaves, stems/tillers, and roots, but with an increased level in leaf tissues compared with stem/tiller tissues (Fig. 4r). These results coincided with the pporRFP fluorescence intensity measurement obtained with leaf tissues (Fig. 4q). All control plants had no pporRFP fluorescence signal or produced PCR amplicons (Fig. 4b, f, j, n, d, h, l, p, Additional file 6: Figure S6e).

## Discussion

### Performance of switchgrass cell suspension cultures

Switchgrass somatic embryogenesis followed by the regeneration of a plant was first described by Dutta Gupta and Conger [25]. However, this work did not report the efficiency of transformation or plant regeneration from single-cell suspension cultures. Mazarei [11] described the establishment, characterization, and applications of cell suspension cultures of switchgrass for the first time in 2011. These authors described three cell type cultures: sandy, fine milky and ultrafine types from genotype Alamo 2. They reported that fine milky type cells were the ones that produced a high amount of protoplasts. However, no further study was conducted on the same type of cells culture or others. Our study developed highly embryogenic single-cell suspension culture systems from friable type II calli using the same MSO medium (Additional file 7: Table S1) in both P32 and P605 ‘Performer’ clones (Fig. 1). The two characterized cell suspension cultures resembled the sandy type suspension culture as described for Alamo 2 above. This type of callus is known for being amenable to produce cell suspension cultures that are competent for somatic embryogenesis and plant regeneration in switchgrass [18, 22, 25] and our study was consistent with these previous findings.

The evaluation of cell growth parameters was also essential for the establishment of our cell suspension culture systems. Methods used for growth characterization of cell suspension culture systems were previously described and utilized [40, 41]. Cell viability and density (Fig. 1 and Additional file 1: Figure S1) showed that cell growth was increased by 60-fold in 14 days, which is as rapid as some other embryogenic liquid systems previously described for various species such as carrot, tomato, Arabidopsis T87, *Sorghum dimidiatum* Stapf, and rice [42–46]. These results were reproduced in repeated experiments.

### *Agrobacterium tumefaciens*-mediated transformation

Switchgrass callus from various explants was first transformed via *Agrobacterium* at the turn of the century [15]. Since that time, *Agrobacterium*-mediated transformation has been improved with respect to various tissue culture methodologies, but has been slow and largely restricted to genotypes from ‘Alamo’ [12, 24], even though reliability and throughput has gradually increased [10]. Li and Qu [18] were the first to report successful transformation of ‘Performer’ using *A. tumefaciens* strain EHA105. The transformation efficiencies obtained from their procedures approached 80%. From that baseline, we developed the two ‘Performer’ lines that appeared to be extraordinarily responsive to tissue culture, transformation, and regeneration. We found the *A. tumefaciens* GV3101 strain appeared to be the best one for the transformation of the embryogenic single-cell suspension cultures and led to high levels of regeneration compared to GV2260, EHA105 or GV3850 (Table 1, Fig. 2, Additional file 2: Figure S2). Moreover, transformation efficiency of up to 85% was observed when the *Agrobacterium* cell density was used at 0.5 DO_600_, and, importantly, transformation efficiencies reached in our system were correlated with stable transformation frequency inoculation and co-culture conditions favoring both T-DNA delivery and recovery of hygromycin B-resistant calli. Previous experiments with various explants of switchgrass and wheat showed that an optimal *Agrobacterium* density of around 0.5 DO_600_ increased the transformation frequency [12, 18, 22, 47]. However, in those studies, the highest transformation frequencies (3.4–90%) were obtained with *A. tumefaciens* strain EHA105, whereas our rates were reproducible and highly obtained with GV3101, followed by GV2260, EHA105 and lastly by GV3850. The higher transformation efficiency achieved in our system makes *A. tumefaciens* strain GV3101 optimal for functional genomics and biotechnological applications in switchgrass. Hence we concluded that there may be a strain × genotype interaction. The combination of embryogenic cell suspension cultures, GV3101, and selected ‘Performer’ genotypes appear to be attractive components for facile and rapid switchgrass transformation and regeneration.

This study included the comparative ability of different ‘Performer’ lines to be transformed with different *A. tumefaciens* strains. Based on previous studies, the choice of switchgrass cultivar was important. In general, the transformation efficiency for selected genotypes from lowland switchgrass using *Agrobacterium*-mediated transformation can reach 56.6–72.8 [18, 20, 48, 49]. However, several attempts to generate transgenic switchgrass using upland switchgrass cultivars resulted in no regenerated plants using upland octoploid cultivar ‘CIR’ [50], only 8% TE for upland tetraploid cultivar ‘Dacotah’ [49] and 7.5% successful transformation rates for upland octoploid cultivar ‘Trailblazer’ [20]. Upland switchgrass lines are generally more recalcitrant to transformation, displaying lower plant regeneration rates, a tighter, stronger shell structure of the callus, and loss of regeneration ability during the transformation process [20, 49, 50]. Our study with switchgrass Performer genotypes, P32 and P605, demonstrated extreme transformation competency for P605 compared to the P32 line (Table 1). These findings might explain why we were not able to successfully reproduce previously published transformation protocol for switchgrass ‘Performer’ cultivar [18] and suggest that *Agrobacterium*-mediated transformation is not only genotype-dependent but also could be clone/line-dependent.

### Regeneration of transgenic switchgrass

**Fig. 5:**
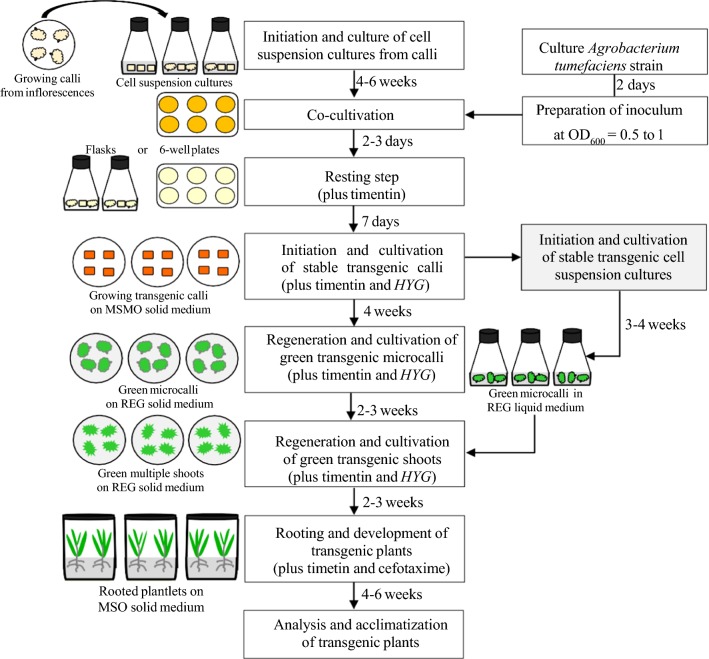
The general workflow of the steps and timeline of a consolidated procedure of *Agrobacterium*-mediated transformation and regeneration in switchgrass ‘Performer’. The expected timeline for all procedures is about 6 to 7 months from the time of initiating cell cultures

Genetic engineering has great potential to improve bioenergy production, and further development of methodology is warranted. A reliable and genotype-independent regeneration system is one highly desirable component. For ‘Performer,’ ‘Alamo,’ and ‘Blackwell GR-63,’ a number of tissue culture and regeneration studies have been performed [12, 18, 22, 23, 48, 49]. These studies reported shoot regeneration efficiencies ranging from 1 to 80% for callus explants grown on a solid medium. However, callus culture regimes, while reliable and amenable to relatively low labor needs, are inefficient, time-consuming, and take 10–12 months to recover transgenic plants. There is a notable absence of reports on the regeneration of switchgrass plants, transgenic or not, from cell suspension cultures. Using our liquid highly embryogenic cell cultures as a source of explants, plant regeneration approaches rates of 100% can be achieved in about 6–7 months (Fig. 3, Table 2, Fig. 5, Additional file 6: Figure S6). Of course, our short timeline is predicated on having plants to establish cell cultures, which adds 3–4 months onto our workflow (Fig. 5). Similar experiments of plant regeneration have been previously reported in *Sorghum dimidiatum*, and *Arabidopsis thaliana* [45, 51]. The authors reported that high-frequency (80%) somatic embryogenesis was obtained from small cell clusters when the culture was initially maintained in liquid medium with a reduced level of 2,4-D (i.e., 0.25 mg l^−1^) followed by the transfer on regeneration medium. In this study, we provided evidence of the use of stable transgenic liquid cell suspension cultures as excellent sources of quasi-explants to rapidly generate transgenic switchgrass. One caveat is we have not determined the lifetime of regenerable cell cultures. While the cell cultures described in this paper are still being maintained (> 2 years), we have not attempted to regenerate transgenic plants over the past year. The system should be adaptable to automation using a liquid handling robot [52, 53] for decreased-labor high-throughput transformation. Automated systems are critical for endowing complex traits via screening gene combinations and circuits in plants, i.e., synthetic biology, a nascent approach in plants [54, 55].

## Conclusions

We report here a new reliable and efficient system for *Agrobacterium*-mediated transformation of switchgrass cv. ‘Performer’ genotypes using cell suspension cultures as sources with the subsequent mass production of transgenic plants. The highly embryogenic cell suspension cultures enabled the recovery of hundreds of putatively transgenic plantlets in 6–7 months. Finally, the optimized new system presented here, substantially improved switchgrass transformation and regeneration potentials, and provides a system for genetic improvement of this vital biofuel feedstock using biotechnological approaches.

## Methods

### Plants, cultures, and transformation

#### Selection of P605 and P32 ‘Performer’ lines

The selection of P605 and P32 lines were performed in two separate research streams. For P605, 1000 ‘Performer’ seeds were sterilized using 100% commercial bleach for 2.5 h on a rotary shaker at 200 rpm, washed three times under non-sterile conditions, and placed at 4 °C overnight. Seeds were sterilized again in 100% commercial bleach and shaken for an additional 80 min. Seeds were then washed with sterile water three times and then left in a sterile hood until seeds were dried. Sterilized seeds were then placed onto LP9 callus induction and maintenance media [12]. A total of 1225 callus segments were transferred onto fresh media after 2 weeks. After 2 months, type II callus was retained and transferred to fresh LP9 media at 2-week intervals. Twenty separate calli yielded at least 90% regeneration frequencies and the resulting plants were ultimately grown in pots in a greenhouse. At the E5 developmental stage, inflorescences were excised, and cultured on MSB media, then transferred to LP9 media after 2 weeks, then lines with type II callus were retained, and regenerated as before. After additional rounds of selection, P605 was chosen as the optimal line for type II callus production and regeneration.

Performer 32 was selected by screening 1100 ‘Performer’ seeds, which were surface sterilized with 70% EtOH, which was decanted and replaced with 100% commercial bleach and 0.1% Tween 20, then shaken at 119 RPM for 2.5 h. The solution was replaced three times with sterile water rinses of 2 min each. The bleach decanted and three sterile water rinses were performed for 2 min each. After blotting the seeds dry, they were cultured on MS-D5-B1 medium for 3 weeks in the dark at 27 °C, then subcultured at 3-week intervals to induce the growth of embryogenic callus [19]. At the end of the first subculture (3 weeks) any genotypes that did germinate or produce callus were discarded. After two additional subcultures, there was 120 calli that produced type II calls, which were then bulked and remaining genotypes were plated again and allowed to grow for three additional weeks. At 6 weeks, genotypes (seed-derived calli) were screened for the production of type II callus [19]. Genotypes that did not produce friable type II callus or produced little to no callus were discarded. Callus of genotypes producing type II callus were bulked for an additional 15 weeks, the fastest-growing callus cultures were selected then regenerated on RSM-B1 media [19]. Regenerated plantlets were grown in culture over 4 weeks in a lighted growth chamber, which included one subculture. A regeneration index [19], resulted in choosing 20 high-performing selections of which P32 was one.

Suspension cultures were initiated from independent calli developed by placing approximatively 1 g of freshly cultured P32 and P605 friable, embryogenic type II calli [56] into 125-ml flasks containing 25 ml of liquid MSO medium [MS supplemented with 9 µM 2,4-diclorophenoxyacetic (2,4-D), 4.4 µM 6-benzylaminopurine (BAP)], pH 5.8 [27, 57] (Additional File 7: Table S1). Cell suspension cultures were maintained in the liquid medium in the dark at 25–28 °C on a rotary shaker at 120 rpm and were subcultured at tenfold dilution with fresh medium every 2 weeks for 4 weeks before any further experiments. The first generation of P32 or P605 cell suspension cultures were established by pipetting the supernatant of 2-week-old cells and then subcultured at fivefold dilution into 250-ml flasks containing 40 ml fresh MSO medium every 2 weeks for 4 weeks. The second generation of P32 or P605 cell suspension cultures was subsequently subjected to the analysis of cell growth and viability, *A. tumefaciens*-mediated transformation and plant regeneration.

#### Cell suspension cultures

Once the P32 and P605 cell suspension cultures were established, growth was measured by using two parameters: the density of cell cultures was determined by evaluating the fresh weight (FW) and the loss of weight by dissimilation (LWD) of cell suspension cultures over 14 days [40, 41]. The fresh weight (FW) method requires harvesting cells to determine the cell density. To sediment cells, 1 ml of cell suspension was harvested and placed in a pre-weighed 1.5-ml Eppendorf tube and centrifuged. The supernatant was removed and then the fresh weight was measured every 2 days over a 14 days period. For LWD, 250 ml flasks containing either only medium or cells in suspension culture of equal 50 ml closed with silicon cap (Chemglass Life Sciences, Vineland, NJ USA) were weighed every 2 days from day 0 to 14. Triplicate control flasks with corresponding enclosures were used to measure the evaporative losses. All flasks were measured at the same time at each time point. We equated differential weights with cell growth after accounting for evaporation.

#### Cell viability

The viability of either P32 or P605 cell suspension cultures was examined using a fluorescein diacetate (FDA, Cat #: 191660050, Acros Organic) staining assay [58]. For staining, 1 ml of cells were gently mixed with an equal volume of 0.05 mg ml^−1^ FDA working solution and incubated for 5 min in the dark at 25 °C. Viable cells exhibiting a bright green fluorescence were observed and scored under an Olympus BX51 epifluorescence microscope (Olympus, America, Melville, NY). Representative images were taken using a confocal Leica TCS SP8 microscope (http://neuronet.utk.edu/utkresources.php) by exciting FDA with the 488 nm and detected via a 505- to 530-nm bandpass filter. Green cells were scored using ten fluorescent images, and the viability was determined as a percentage fraction of surviving cells calculated by dividing the number of viable green cells by the total count of cells multiplied by 100.

#### *Agrobacterium tumefaciens* strains, culture and plasmid vector

The transformation was performed using four *A. tumefaciens* strains, GV3101 [59], GV2260 [60], EHA105 [61] and GV3850 [60, 62]. For culture, a single colony of each *Agrobacterium* strain harboring the expression vector construct pANIC-10A, was suspended in 5 ml yeast extract and peptone (YEP) medium [63] supplemented with the appropriate antibiotics: 50 µg ml^−1^ gentamicin plus 10 µg ml^−1^ rifampicin for GV3101, 10 µg ml^−1^ rifampicin for GV2260, EHA105 and GV3850, and 50 µg ml^−1^ kanamycin (for plasmid selection). After 24 h, 50 µl of the above culture was transferred to 50 ml YEP medium containing appropriate antibiotics and incubated at 200 rpm on a rotary shaker (MAXQ6000, Thermo Scientific) at 28 °C until the culture reached optical density OD_600_ = 1. After 2 days of growth, the cultures were centrifuged at 3000 rpm (Sorvall Legend XTR centrifuge, Thermo Scientific) for 5 min. The pellet was then washed twice with MSMO medium [64], (Additional file 7: Table S1) supplemented with 100 µM acetosyringone [12] for P32 and P605 cell suspension cultures. The final bacterial pellet was diluted with fresh modified MSMO medium (Additional file 7: Table S1) to adjust the inoculum concentration to final densities 0.5 and 1.0 OD_600_. Each strain harbored the same binary vector pANIC-10A [62, 65] that carried the switchgrass polyubiquitin 1 promoter and intron (*PvUbi1*), which drives the expression of *Porites porites* red fluorescent protein coding region (*pporRFP*) and hygromycin B phosphotransferase coding region (*HYG*) regulated by switchgrass polyubiquitin 2 promoter and intron (*PvUbi2*). The *HYG* gene confers resistance to the hygromycin antibiotic.

#### *Agrobacterium*-mediated stable transformation of liquid cell suspension cultures

The transformation procedure was conducted using a method developed in our laboratory, modified from previous protocols [12, 18] and an *Arabidopsis thaliana* suspension culture transformation protocol (VIB, ABRC Ohio State University). The P32 and P605 cell suspension cultures were transformed according to an *A. tumefaciens*-mediated DNA delivery method [66]. The transformation was performed using either the culturing MSO medium or modified MSMO medium. Before co-cultivation, cell suspension cultures were preconditioned for 24 h in a liquid MSMO medium, and then an aliquot of 3 ml (80 mg ml^−1^ of fresh weight cells) was mixed with each bacterial inoculum at two different concentrations 0.5 and 1.0 OD_600_. The samples were co-cultivated under gentle agitation for 2–3 days in the dark and kept at 25 ± 2 °C. After co-cultivation, competent P32 or P605 cells suspension cultures were washed three times with MSMO medium containing 400 mg l^−1^ timentin [12] to eliminate bacteria, and then transferred to fresh medium and kept under gentle agitation in the dark for 7 days. At that point, switchgrass cells were spread on MSMO solid medium supplemented with 400 mg l^−1^ timentin and 50 mg l^−1^ hygromycin and cultured in the dark for 1 month. Then, hygromycin B-resistant switchgrass calli were scored and screened for positive pporRFP fluorescent protein expression. The hygromycin B-resistant calli were used either directly to generate shoots and plants, or maintained to establish stable transgenic liquid cell lines as described before by Wang [67], and subsequently used for the regeneration of shoots and plants.

### Regeneration of shoots and plants

The regeneration of transgenic and non-transgenic shoots and plants for both P32 and P605 lines was performed in two ways, using either transgenic calli or transgenic liquid cell cultures. Both methods used were modified from methods previously described [12, 18, 24]. To generate green shoots from transgenic calli, embryogenic calli were subcultured every 2 weeks for 1 month, placed in REG solid medium (Additional file 7: Table S1), and kept under cool white fluorescent light (140 µmol m^−2^ s^−1^) with a photoperiod of 16/8 h (light/dark) at 25 °C in a growth chamber. 2–3 weeks later, 10 pieces of green micro-calli were placed on Petri dishes to generate shoots. Twenty to 30 pieces transgenic micro-calli were used per replicate and for each line; the experiment was repeated three times. Transgenic and non-transgenic shoots were also regenerated from liquid cell culture lines established from 1 g of 1-month-old calli. Transgenic or non-transgenic cell cultures were initiated and cultured for 4 weeks in REG liquid media containing 50 mg l^−1^ hygromycin B and 400 mg l^−1^ timentin. A dilution of 1–5 two-week-old transgenic cell suspension cultures was used to generate transgenic green micro-calli in liquid for 2 weeks. At this point, 100 green transgenic and non-transgenic shoots generated using either method were transferred to magenta vessels (5 shoots per box) (GA-7, Sigma-Aldrich) containing MSO medium supplemented with 250 mg l^−1^ cefotaxime [68] and plantlets were allowed to develop and root for 4–6 weeks. Hundreds of regenerating transgenic and non-transgenic rooted plantlets (plantlets having shoots and roots) were transferred to the soil for growth and development, and acclimatization in the growth chamber. Regenerating transgenic and non-transgenic plants that appeared to be morphologically indistinguishable from seed-grown plants were scored and screened for the presence of transgene expression before being placed in potting media in pots to assess their growth and development in the greenhouse. The *T*_0_ plants grown in soil were subsequently analyzed after 2–3 months as described below.

### Microscopy and spectrofluorometry

The analysis of transgenic calli, cells or plants were performed as previously described [62]. Fluorescence microscopy was carried out using the tdTomato filter set: 554-nm excitation and 581-nm emission wavelength with an Olympus stereo microscope model SZX12 (Olympus America, Center Valley, PA, USA) (for callus imaging) and an Olympus BX51 epifluorescence (for cell imaging). Confocal microscopy images were produced using a confocal Leica TCS SP8 microscope. The samples were excited with a 543 nm HeNe laser and fluorescence emission was collected from 590 to 610 nm for pporRFP. Fluorescence intensity was measured using a spectrofluorometry according to methods described by Millwood [69] with a Fluorolog^®^-3 system (Jobin-Yvon and Glen Spectra, Edison, NJ, USA). Triplicate spectra/peak emission absorbance was adjusted by removing the background signal from corresponding controls used for each sample. For each sample, the youngest fully expanded leaf from *T*_0_ lines was chosen to measure the intensity of fluorescence in non-transgenic control and putatively transgenic plants.

### PCR analysis

PCR analysis was used to assess transgenicity of putative *T*_0_ plants [18, 70]. The genomic DNA (gDNA) was isolated from leaf tissues harvested from each putative *T*_0_ line as previously described by Edwards [71]. For all PCR reactions, an EconoTaq Plus Green 2X Master Mix (Lucigen) with the Eppendorf Master Cycler Pro S (USA Scientific) were used as previously described [72]. Both *HYG* and *pporRFP* were amplified using established primer sets (Additional file 8: Table S2).

### Transcript analysis by real-time RT-PCR

Transcript abundance was estimated by real-time RT-PCR analysis as described by Ondzighi-Assoume [72] with few modifications. Total RNA was isolated from leaf, stem and root tissues harvested from 2-month-old transgenic and non-transgenic P32 and P605 plants. The isolation of RNA was performed using the Qiagen RNeasy Plant Mini Kit (Qiagen) and subsequently treated with Turbo DNase-free (Ambion) to remove genomic DNA contamination, and then subject to quantitative PCR with the ABI QuantStudio6 Flex Real-time PCR system (Applied Biosystems, ThermoFisher Scientific). Data were collected and analyzed according to the ΔΔCT method and normalized to the geometric mean of the expression of two housekeeping genes, *P. virgatum* L. ACTIN2 (*PvACT*) and *P. virgatum* L. UBIQUITIN (*PvUBQ*) [65] with the Quanta Studio™ 6 and 7 Flex System Software. Nucleotide sequences of primers used are listed in the Additional file 8: Table S2.

### Statistical analysis

Statistical analysis for all the experiments was performed using GraphPad Prism software (GPW6) [72]. Data were plotted as the mean ± standard error (SE) of three biological replicates. For the analysis of all data, the significance of differences between different groups was assessed using ANOVA and Tukey’s multiple comparisons test at *p* ≤ 0.05.

## Supplementary information

**Additional file 1: Figure S1.** Dissimilation growth curve of P32 and P605 cell suspension cultures.

**Additional file 2: Figure S2.** Comparison of the transformation efficiency of four *Agrobacterium* strains to transform P32 and P605 cell suspension cultures.

**Additional file 3: Figure S3.** Auto-fluorescence controls for pporRFP fluorescence signal specificity in transgenic P32 and P605 cell suspension cultures.

**Additional file 4: Figure S4.** Characterization of stable transgenic P32 and P605 cell suspension cultures expressing the pporRFP fluorescent protein.

**Additional file 5: Figure S5.** Regeneration of the root system in 6-week-old (post-shooting) transgenic P32 and P605 plantlets.

**Additional file 6: Figure S6.** Phenotype and PCR analysis of regenerated T_0_ transgenic and non-transgenic P32 and P605 plants.

**Additional file 7: Table S1.** Media used for the establishment, characterization, transformation, and plant regeneration for P32 and P605 cell suspension cultures.

**Additional file 8: Table S2.** Sequences of *HYG*, *pporRFP*, and housekeeping gene primers used for PCR and qRT-PCR.

## Data Availability

All data generated or analyzed during this study are included in this published article (and its additional information files).

## Abbreviations

2,4-D: 2,4-dichlorophenoxyacetic acid
BAP: 6-benzylaminopurine
FDA: fluorescein diacetate
MS: Murashige and Skoog medium
MSB: MS supplemented with BAP and sucrose
MSO: MS supplemented with BAP and maltose
MSMO: MS supplemented with sucrose
REG: regeneration medium
P32: *Panicum virgatum* var. Performer32
P605: *Panicum virgatum* var. Performer605
