# Supplementary material for: Embryogenic cell suspensions for high-capacity genetic transformation and regeneration of switchgrass (Panicum virgatum L.)
Source: Biotechnol Biofuels. 2019 Dec 16;12:290. doi: 10.1186/s13068-019-1632-3 (PMC6913013; doi:10.1186/s13068-019-1632-3)
Supplement: Supplementary file 1 — Additional file 1: Figure S1. Dissimilation growth curve of P32 and P605 cell suspension cultures. [file 13068_2019_1632_MOESM1_ESM.docx]

**Additional file 1**

**
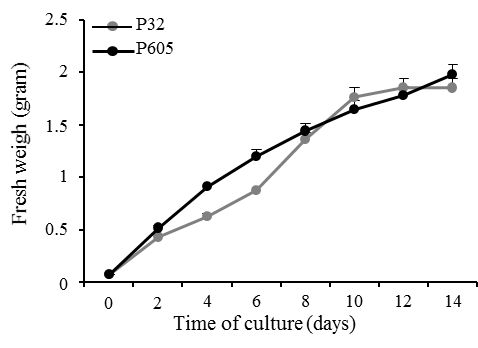
**

**Fig. S1**. Dissimilation growth curve of P32 and P605 cell suspension cultures.

Growth characteristics of P32 (gray circles graph) and P605 (black circles graph) cell suspension cultures as evaluated by loss weigh by dissimilation (LWD) over time of the culture. Experiments were done in triplicate. Error bars represent the mean ± standard error (SE).
