# Supplementary material for: Embryogenic cell suspensions for high-capacity genetic transformation and regeneration of switchgrass (Panicum virgatum L.)
Source: Biotechnol Biofuels. 2019 Dec 16;12:290. doi: 10.1186/s13068-019-1632-3 (PMC6913013; doi:10.1186/s13068-019-1632-3)
Supplement: Supplementary file 2 — Additional file 2: Figure S2. Comparison of the transformation efficiency of four Agrobacterium strains to transform P32 and P605 cell suspension cultures. [file 13068_2019_1632_MOESM2_ESM.docx]

**Additional file 2**

a

a

a

b

d

0

20

40

60

80

a

GV3101

GV2260 EHA105 GV3850

Resistant calli (%)

P32 P605

c

d

P605

P32

**^a^** 100

**b**

GV3101 GV2260 EHA105 GV3850

**Fig. S2**. Comparison of the transformation efficiency of four *Agrobacterium* strains to transform P32 and P605 cell suspension cultures. **a** Efficiencies of the transformation of strains GV3101, GV2260, EHA105, and GV3850 at OD_600_ = 1.0. Transformation efficiency was evaluated by scoring hygromycin B resistant-calli with pporRFP fluorescence signal after one month of cultivation on hygromycin selection medium. The data depicted in the graph represent the average of three replications of the transformation event. Gray columns represent P32 calli. Black columns represent P605 calli. Different letters denote a statistically significant difference among means at a p-value < 0.05 using a one-way ANOVA (Tukey's test). Bars represent SEM (n = 10 plates). **b** Digital photos of representative one-month-old hygromycin resistant-calli derived from P32 and P605 cell suspension cultures transformed with *A. tumefaciens* strain GV3101, GV2260, EHA105 or GV3850 at the same co-cultivation time with *A. tumefaciens* cells at the density OD_600_ = 0.5.
