# Supplementary material for: Embryogenic cell suspensions for high-capacity genetic transformation and regeneration of switchgrass (Panicum virgatum L.)
Source: Biotechnol Biofuels. 2019 Dec 16;12:290. doi: 10.1186/s13068-019-1632-3 (PMC6913013; doi:10.1186/s13068-019-1632-3)
Supplement: Supplementary file 3 — Additional file 3: Figure S3. Auto-fluorescence controls for pporRFP fluorescence signal specificity in transgenic P32 and P605 cell suspension cultures. [file 13068_2019_1632_MOESM3_ESM.docx]

**Additional file 3**


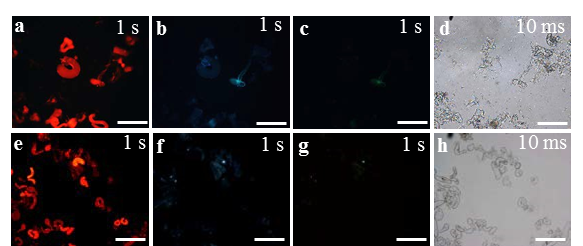


**Fig. S3**. Auto-fluorescence controls for pporRFP fluorescence signal in transgenic P32 and P605 cell suspension cultures. **a** to **h** Epifluorescence micrographs showing 14-day-old stable transgenic P32 (**a** to **d**) and P605 (**e** to **h**) cells expressing the pporRFP fluorescent fusion protein. **a** and **e** the pporRFP fluorescence signal of cells visualized under tdTomato filter set (excitation at 554 nm, emission wavelength at 581 nm [red]). **b** and **f** Auto-fluorescence of cells visualized under a DAPI filter set (excitation at 358 nm and emission at 461 nm [blue]). **c** and **g** Auto-fluorescence signal of cells visualized under a FITC filter set (excitation at 488 nm, emission at 505–530 nm [green]). **d** and **h** Bright-field images of cells. Exposure times are indicated on the images. In both, DAPI and FITC filter sets used, no fluorescence signal was observed. These control observations confirmed the specificity of the pporRFP fluorescence protein expression for the transgenic cells studied Bars = 10 µm **a** to **f**.
