# Supplementary material for: Embryogenic cell suspensions for high-capacity genetic transformation and regeneration of switchgrass (Panicum virgatum L.)
Source: Biotechnol Biofuels. 2019 Dec 16;12:290. doi: 10.1186/s13068-019-1632-3 (PMC6913013; doi:10.1186/s13068-019-1632-3)
Supplement: Supplementary file 4 — Additional file 4: Figure S4. Characterization of stable transgenic P32 and P605 cell suspension cultures expressing the pporRFP fluorescent protein. [file 13068_2019_1632_MOESM4_ESM.docx]

**Additional file 4**

**
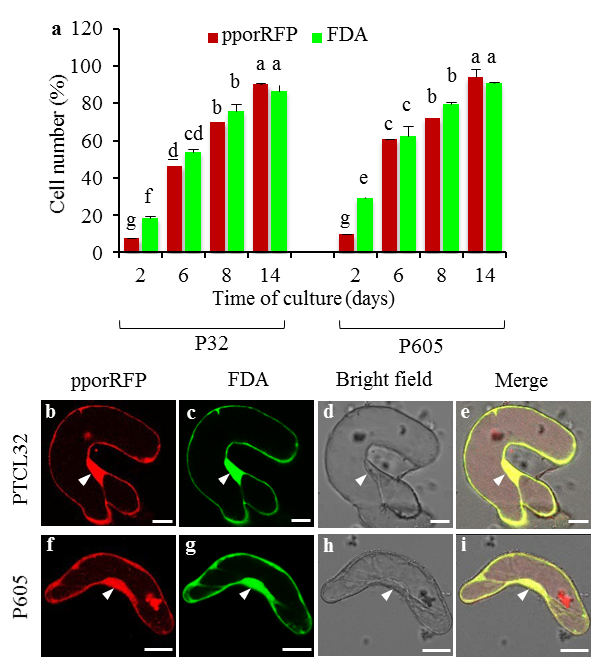
**

**Fig. S4**. Characterization of stable transgenic P32 and P605 cell suspension cultures expressing the pporRFP fluorescent protein. **a** Quantification of pporRFP fluorescent and viable transgenic cells of P32 and P605 lines grown within 14 days. Red columns represent the percentage of cells expressing pporRFP. Green columns represent the percentage of viable cells. All viable fluorescent cells quantifications were normalized to the total number of cells in culture. Error bars represent the mean ±SE of three biological replicates, and different letters denote a statistically significant difference among means at a p-value < 0.05 according to One-way ANOVA (Tukey's test). **b** to **i** Confocal micrographs showing the subcellular localization of pporRFP fluorescent fusion protein in 7-day-old stable transgenic P32 (**b** to **e**) and P605 (**f** to **i**) within the cytoplasmic space. **b** and **f** PporRFP fluorescence signal in red. C and G, FDA staining in green. **d** and **h** Bright filed images. **e** and **i** Merge images. White arrowheads indicate the nuclear localization of pporRFP and FDA. Bars = 10 µm **b** to **i**.
