# Supplementary material for: Embryogenic cell suspensions for high-capacity genetic transformation and regeneration of switchgrass (Panicum virgatum L.)
Source: Biotechnol Biofuels. 2019 Dec 16;12:290. doi: 10.1186/s13068-019-1632-3 (PMC6913013; doi:10.1186/s13068-019-1632-3)
Supplement: Supplementary file 5 — Additional file 5: Figure S5. Regeneration of the root system in 6-week-old (post-shooting) transgenic P32 and P605 plantlets. [file 13068_2019_1632_MOESM5_ESM.docx]

**Additional file 5**

P32

P605

**a**

**b**

**c**

**d**

**Fig. S5**. Plant regeneration in early (6-week-old post-shooting) transgenic P32 and P605 events. **a** and **b** Photos of regenerating roots of transgenic P32 plantlets. **b** High magnification of the bottom of the magenta box of panel **a**. **c** and **d** Regenerating roots of transgenic P605 plantlets. **d** High magnification of the bottom of the magenta box of panel **c**.
