# Supplementary material for: Embryogenic cell suspensions for high-capacity genetic transformation and regeneration of switchgrass (Panicum virgatum L.)
Source: Biotechnol Biofuels. 2019 Dec 16;12:290. doi: 10.1186/s13068-019-1632-3 (PMC6913013; doi:10.1186/s13068-019-1632-3)
Supplement: Supplementary file 6 — Additional file 6: Figure S6. Phenotype and PCR analysis of regenerated T0 transgenic and non-transgenic P32 and P605 plants. [file 13068_2019_1632_MOESM6_ESM.docx]

**Additional file 6**

Mk NC_1_ NC_2_

P32

P605

pANIC 10A

*HYG*

*pporRFP*

1 2 3 4 5 6 ^NC'^_1_ ^NC'^_1_ ^1'^

2' 3' 4'

5' 6' PC1 PC2

P605

P32

**a**

**b**

**c**

**d**

**e**


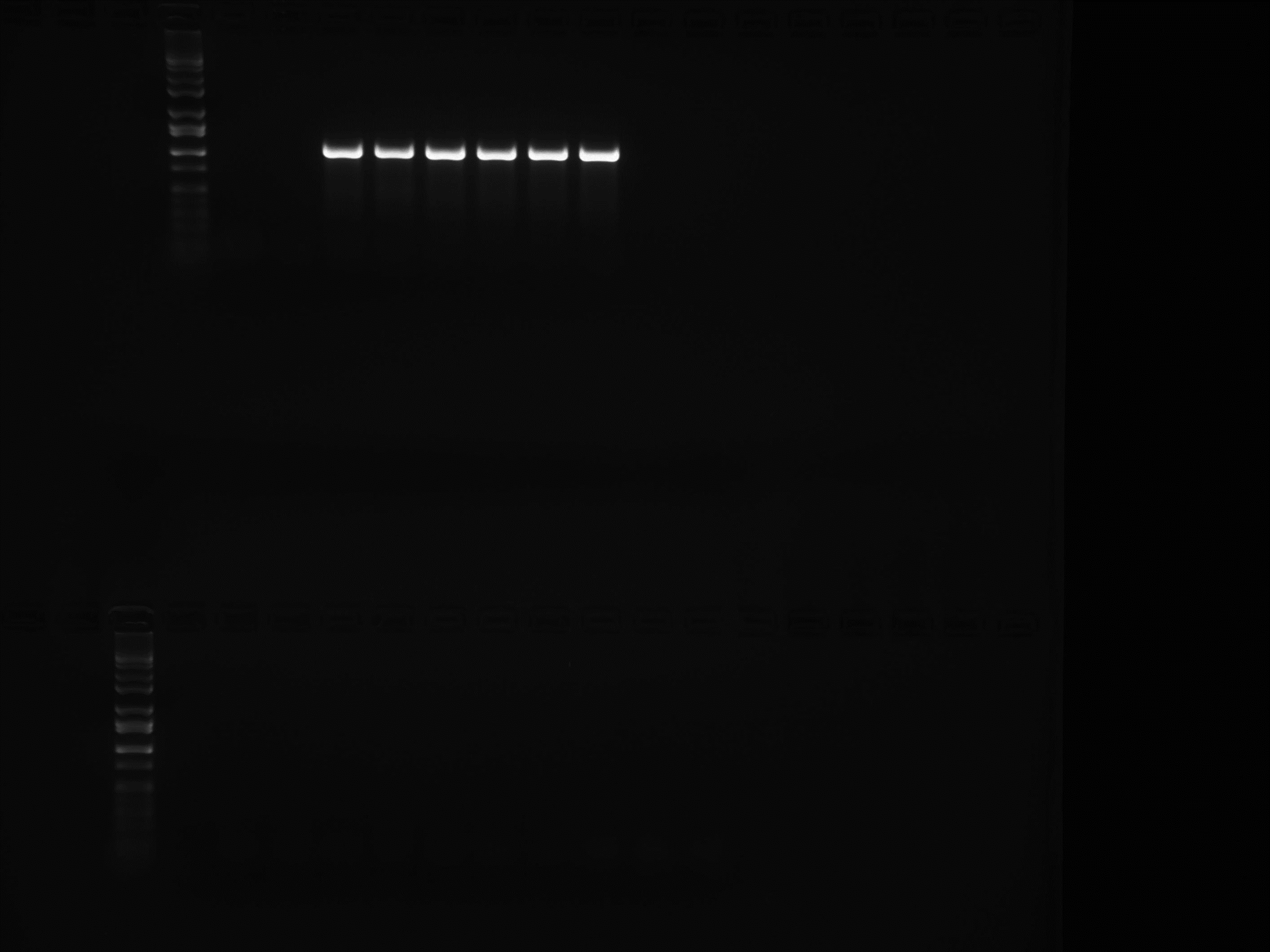

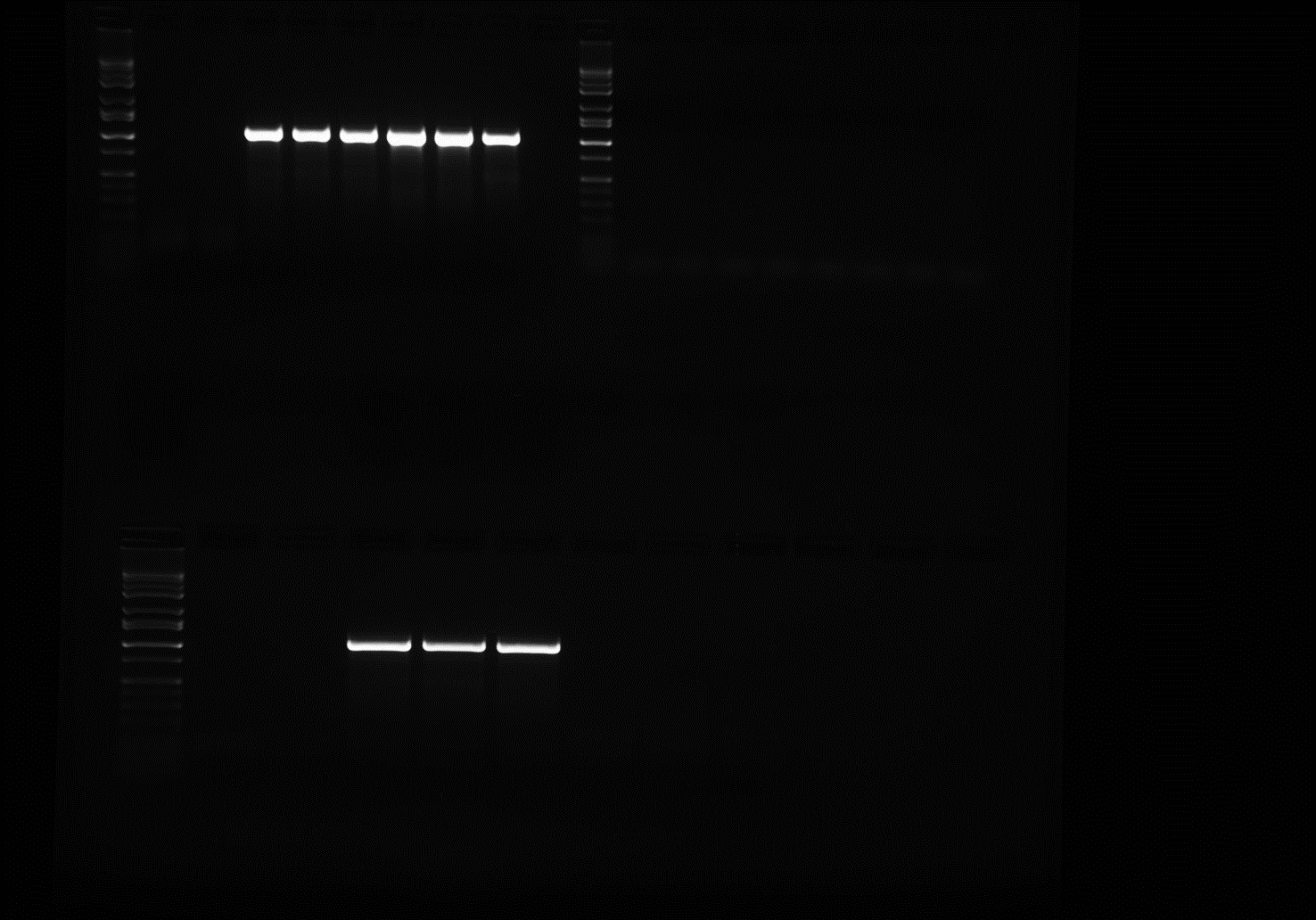

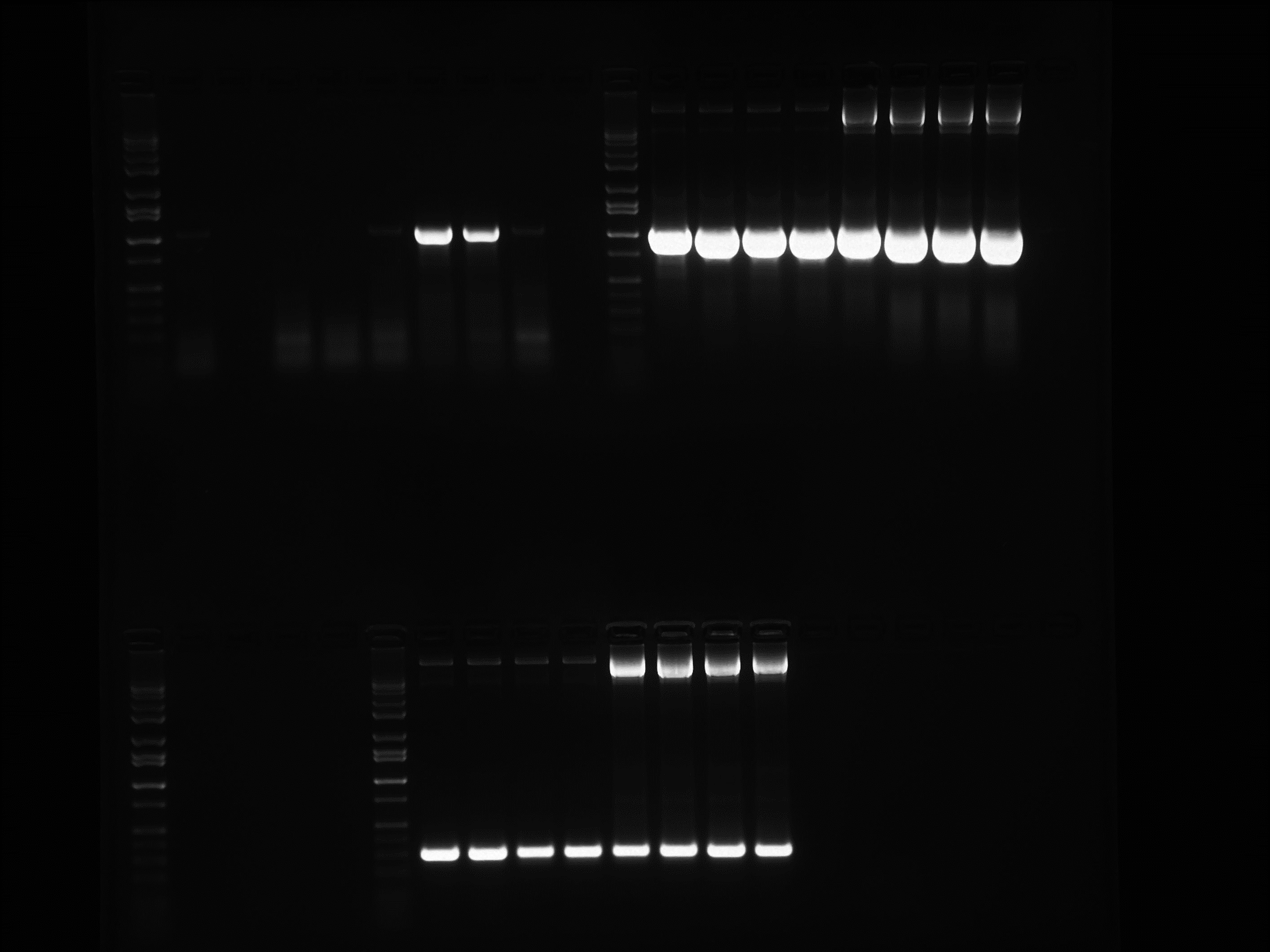


**C2 C4 T1 T2 T3 T4 T5 T6**


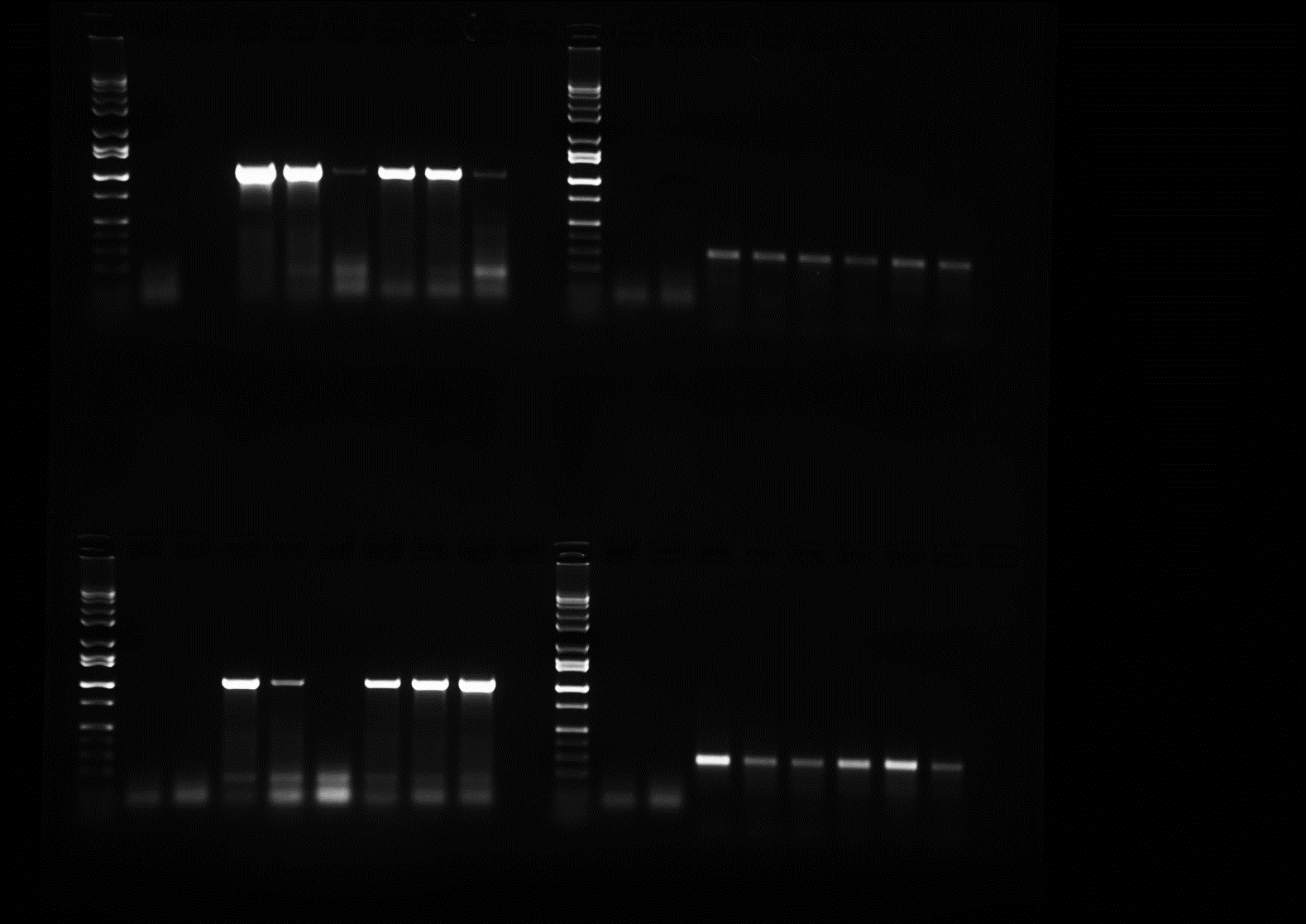

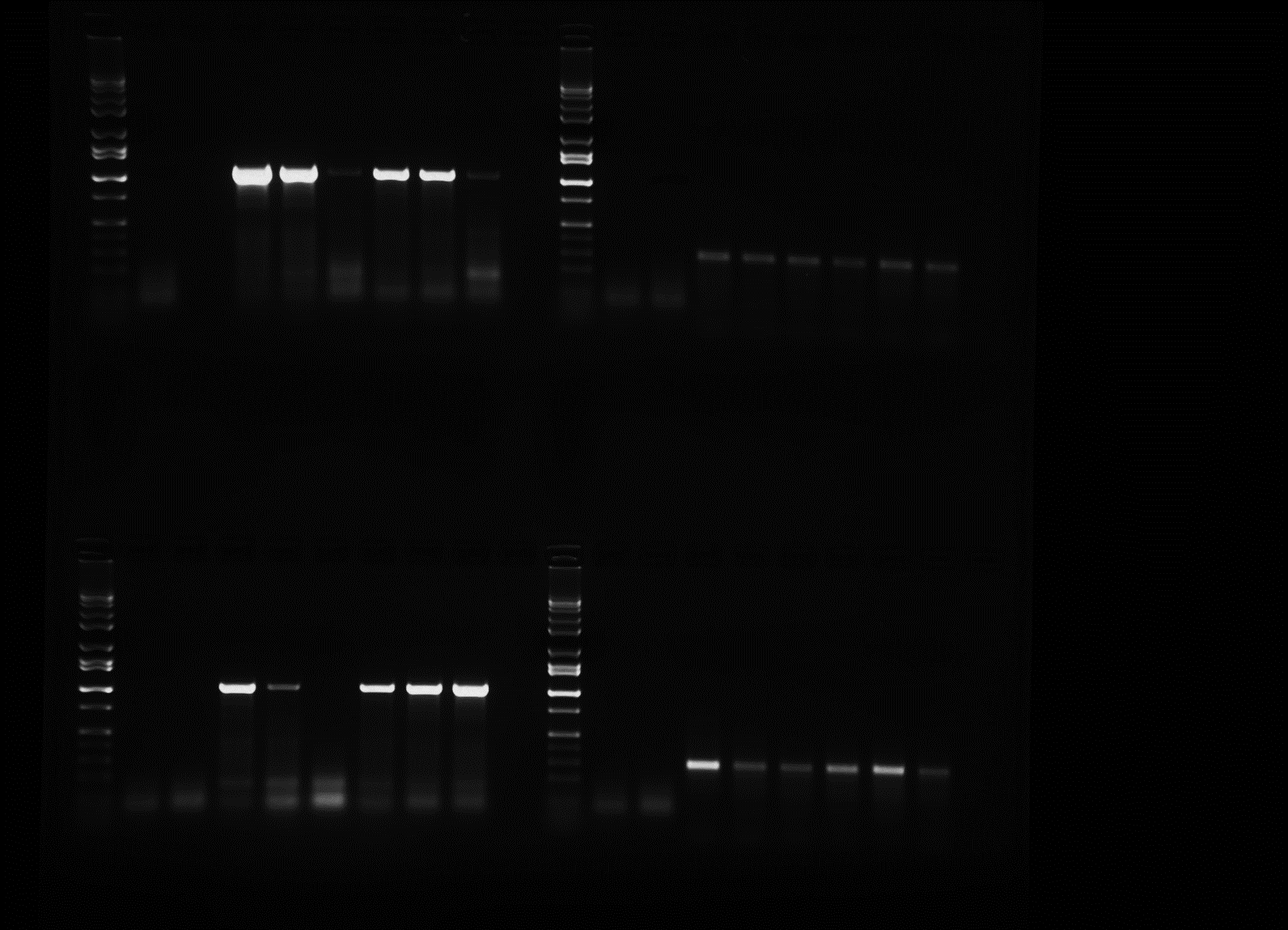

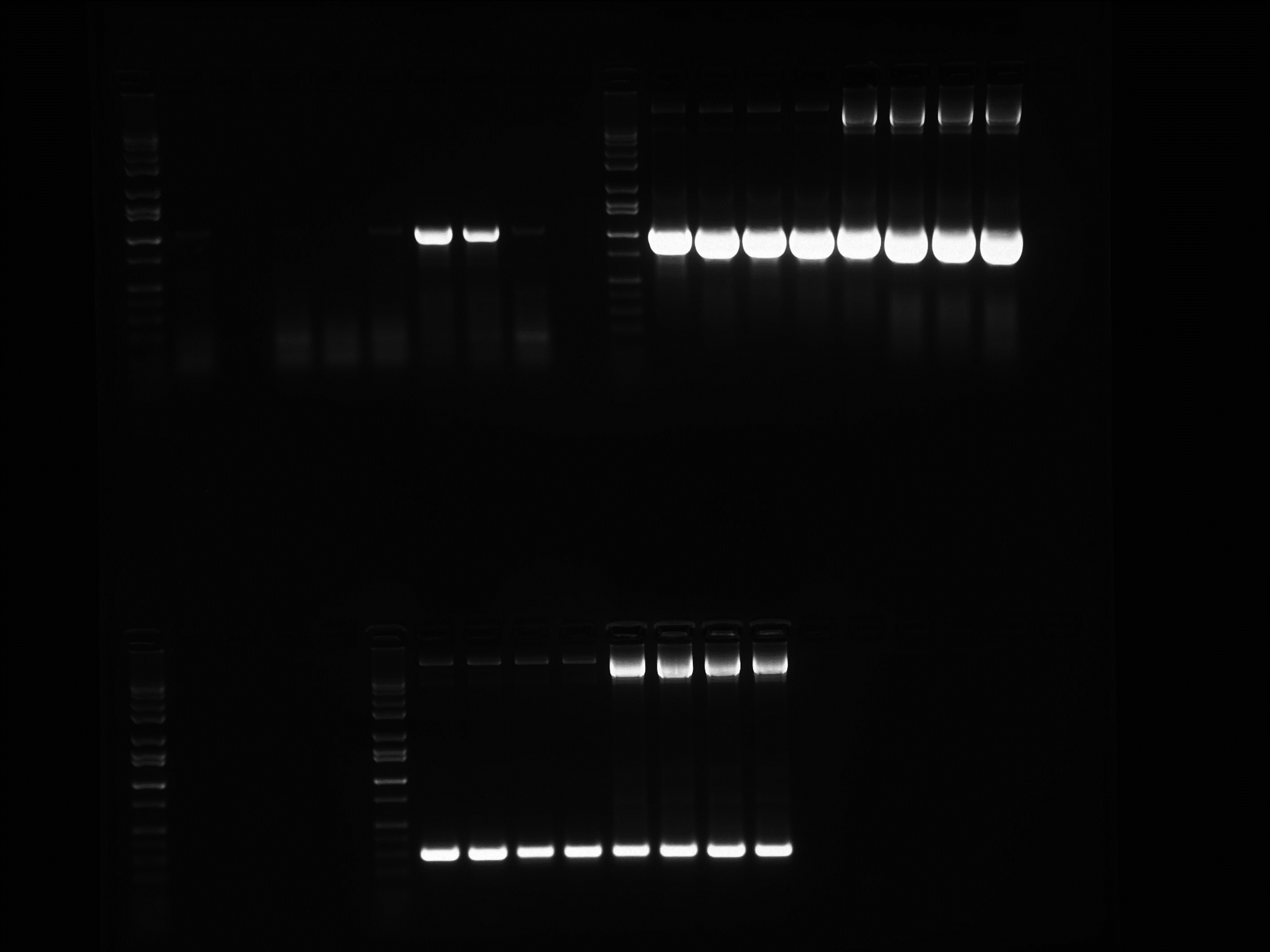


**Fig. S6**. Phenotype and PCR analysis of regenerated T_0_ transgenic and non-transgenic P32 and P605 plants. **a** to **d** Images of healthy 2-month-old regenerated T_0_ transgenic and non-transgenic P32 (**a** and **b**) and P605 (**c** and **d**) plants growing on soil. **e** PCR analysis of transgenic and non-transgenic P32 and P605 plants showing the presence of *HYG* and *pporRFP* genes. Mk. Hi-Lo^TM^ DNA marker, NC and NC' are negative controls of the genomic DNA (gDNA) extracted from T_0_ non-transgenic P32 and P605 plants respectively (NC_1_ and NC_2_ or NC'_1_ and NC'_2_ are two set of four individual controls plant of P32 and P605 transformed with *A. tumefaciens* alone respectively), PC1 and PC2 are positive controls of two different concentrations (50 and 100 ng) of pANIC 10A plasmid DNA, 1-6 and 1'-6' are amplified gDNAs extracted from six individual T_0_ transgenic plants of each P32 and P605 line respectively. Amplicon sizes (indicated by the arrows) are 1 kb and 0.35 kb for *HYG* and *pporRFP* genes respectively.
