# Supplementary material for: Embryogenic cell suspensions for high-capacity genetic transformation and regeneration of switchgrass (Panicum virgatum L.)
Source: Biotechnol Biofuels. 2019 Dec 16;12:290. doi: 10.1186/s13068-019-1632-3 (PMC6913013; doi:10.1186/s13068-019-1632-3)
Supplement: Supplementary file 7 — Additional file 7: Table S1. Media used for the establishment, characterization, transformation, and plant regeneration for P32 and P605 cell suspension cultures. [file 13068_2019_1632_MOESM7_ESM.docx]

**Additional file 7**

**Table S1**. Media used for the establishment, characterization, transformation, and plants regeneration for cell suspension cultures.

**
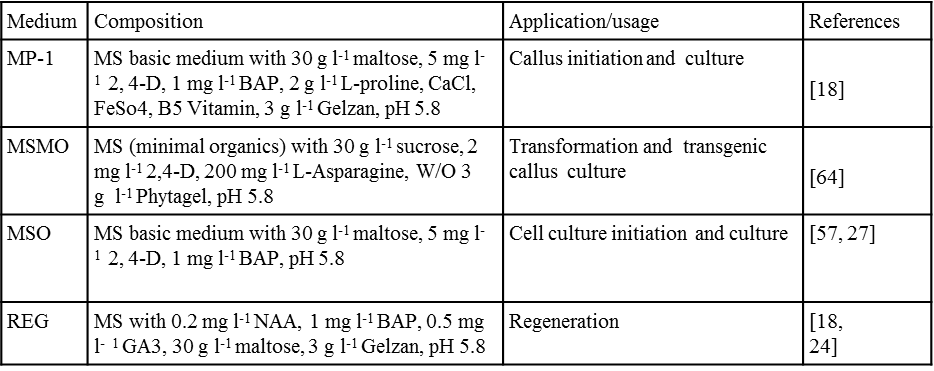
**
