# Supplementary figures and images for: Embryogenic cell suspensions for high-capacity genetic transformation and regeneration of switchgrass (Panicum virgatum L.)
Source: Biotechnol Biofuels. 2019 Dec 16;12:290. doi: 10.1186/s13068-019-1632-3 (PMC6913013; doi:10.1186/s13068-019-1632-3)

**Additional file 8**

**Table S2**. Sequences of *HYG*, *pporRFP* and housekeeping gene primers.

**
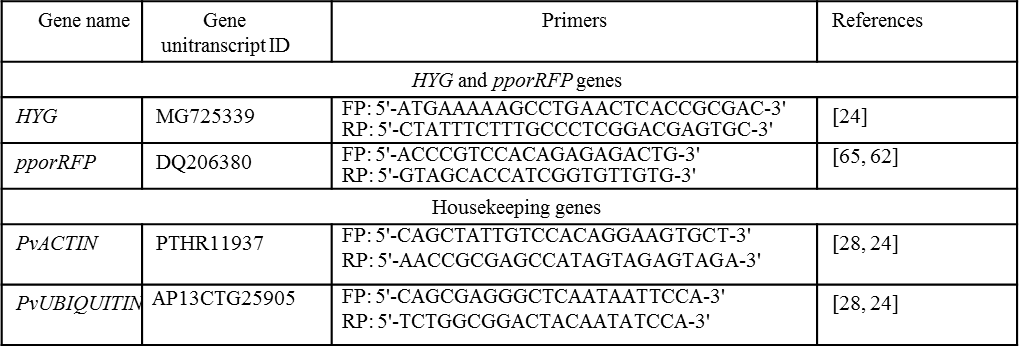
**

Supplement: Supplementary file 8 — Additional file 8: Table S2. Sequences of HYG, pporRFP, and housekeeping gene primers used for PCR and qRT-PCR. [file 13068_2019_1632_MOESM8_ESM.docx]
